# Inflammatory cytokines in highly myopic eyes

**DOI:** 10.1038/s41598-019-39652-x

**Published:** 2019-03-05

**Authors:** Jianshu Yuan, Shanjun Wu, Yuewen Wang, Suqi Pan, Pengyun Wang, Lingyun Cheng

**Affiliations:** 1 Ningbo Eye Hospital, 855 Min An Road, Ningbo, Zhejiang 315040 China; 2 Jacob’s Retina Center at Shiley Eye Institute, Department of Ophthalmology, University of California San Diego, 9415 Campus Point Drive, La Jolla, CA 92037-0946 USA

## Abstract

Currently, myopic retinopathy is the most common irreversible blinding disease but its pathophysiology is not completely clear. A cross-sectional, observational study was conducted in a single center to analyze aqueous samples from highly myopic eyes (axial length >25 mm, n = 92) and ametropic or mild myopic eyes (n = 88) for inflammatory cytokines. Vascular endothelial growth factor (VEGF), Interleukin 6 (IL-6), and matrix metalloproteinase-2 (MMP-2) were measured using an enzyme-linked immunosorbent assay. IL-6 and MMP-2 were significantly higher in the highly myopic eyes than in the non-high myopic eyes (IL-6: 11.90 vs. 4.38 pg/mL, p < 0.0001; MMP-2: 13.10 vs. 8.82 ng/mL, p = 0.0003) while adjusting for age, gender, and intraocular pressure. There was a significant positive association between levels of IL-6 and MMP-2 in aqueous humor and the axial lengths of the eye globes (IL-6, β = 0.065, p < 0.0001, n = 134; MMP-2, β = 0.097, p < 0.0001, n = 131). Conversely, VEGF in aqueous humor was significantly lower in the highly myopic eyes than in the non-high myopic eyes (45.56 vs. 96.90 pg/mL, p < 0.0001, n = 153) while age, gender, and intraocular pressure were adjusted. The results suggest that low-grade intraocular inflammation may play an important role in the development and progression of high myopia and myopic retinopathy.

## Introduction

Myopia is a very common refractive disorder of the eye. Mild or moderate myopia usually stabilizes within the third decade of life without pathological changes of the retina later in life. However, there are many patients whose refractive error and eye structure experience a progressive change over their whole lifetime. These changes include elongation of the eye globe axis, stretching of the eye wall, degenerative changes such as geographic atrophy of the retina and choroid, and choroidal neovascularization at the macular region. These pathological changes occur later in life (fifth decade and later) and can cause significant visual loss and disability^1–5^. Large population-based studies have shown that high myopia is the leading eye disorder to cause visual disability^4–6^, only second to cataract in the Asian population^6,7^. Myopia has become a worldwide health issue afflicting 1.4 billion people worldwide and a projected 4.7 billion people will have myopia (49.8% of the world population) by 2050. Of those, 163 million have high myopia with progressive eye globe elongation and develop blinding myopic retinopathy^8^.

Currently there is no ideal therapy to halt the progressive elongation of axial length in highly myopic eyes, although posterior scleral reinforcement surgery early in life is being investigated^9,10^. The root cause of this devastating eye disorder is not completely clear; both genetic^11–13^ and environmental factors have been suggested to be at play^14,15^. In daily retina practice, chronic inflammatory chorioretinal diseases are often noted to have a myopic refractive shift over time. With the advent of OCT imaging technology, stretching of the sclera or development of staphyloma can be monitored during treatment and follow-up of some inflammatory ocular disease such as Vogt-Koyanagi-Harada disease (ARVO abstract 3126, Yosuke Harada, on Tuesday, May 5, 2015)^16^. If choroid inflammation can weaken and cause the sclera to stretch, resulting in myopia as seen in Vogt-Koyanagi-Harada disease, it is possible that persistent low-grade chronic inflammation in the retina/choroid could cause progressive stretching of the sclera and axial elongation. Indeed, the data from a large study of chorioretinal inflammatory diseases with fifteen years of follow-up revealed that myopic refractive shift was present in every inflammatory disease entity including multifocal choroiditis (average −2.19 diopters), punctate inner choroidopathy (average −3.67 diopters), diffuse subretinal fibrosis syndrome (average −1.25 diopters), and multiple evanescent white dot syndrome (average −1.25 diopters)^17^. There may be a connection between myopia (and the associated retinal degeneration) and innate subclinical inflammation in the retina/choroid. The neural retina, as an extension of the vertebrate brain, shares many anatomical and physiological features such as tight endothelial barriers. Several degenerative changes in the brain, such as Alzheimer’s disease and dementia, have been reported to be attributable to chronic inflammation^18–20^. While the retina of highly myopic eyes exhibits clear degenerative changes^5^, it is not yet well explored if the retinal degeneration is related to inflammation.

The association between myopia and subclinical chorioretinal inflammation has rarely been explored and relevant data is in paucity^21,22^. One reason for this is that there is no clinically perceivable inflammation in the retina or choroid of myopic eyes and it is not justified to sample ocular fluid from these patients. Senile cataract extraction in emmetropic and myopic eyes provides an excellent opportunity for us to sample aqueous humor from selected patients. Studies have demonstrated that the levels of drugs or cytokines are positively correlated between the aqueous and vitreous fluid^23,24^. The aim of this study is to investigate if there is indeed a connection between low-grade intraocular inflammation and highly myopic eyes using undiluted aqueous humor samples from a senile cataract population who received cataract extraction at a single eye hospital from January 2016 to October of 2017.

## Materials and Methods

### Study design

We hypothesize that persistent low-grade chronic inflammation may be one of the factors contributing to progressive axial elongation in highly myopic eyes or pathological myopia. Eyes with −8 diopters or worse are at significantly higher risk for developing a myopic retinopathy later in life, including diffuse chorioretinal atrophy, patchy chorioretinal atrophy, and macular atrophy^25^. For this sake, senile cataract patients with a history of high myopia and an axial length greater than 25 mm, or concurrent senile cataract patients without myopia or an axial length of 25 mm or shorter were prospectively selected at the pre-cataract surgery examination with the patients’ signed informed consent. At the start of cataract surgery following retro bulbar anesthesia, the undiluted aqueous humor was collected immediately prior to the intracameral injection of viscoelastic material. The sampled volume was between 90 µL and 120 µL depending on eye size, depth of the anterior chamber, and the procedural safety judged by the surgeon. The ethics committee of the Ningbo Eye Hospital approved the study and all study participants gave their consent before the surgery. This research strictly adhered to the tenets of the Declaration of Helsinki.

### Participants

**Figure 1:**
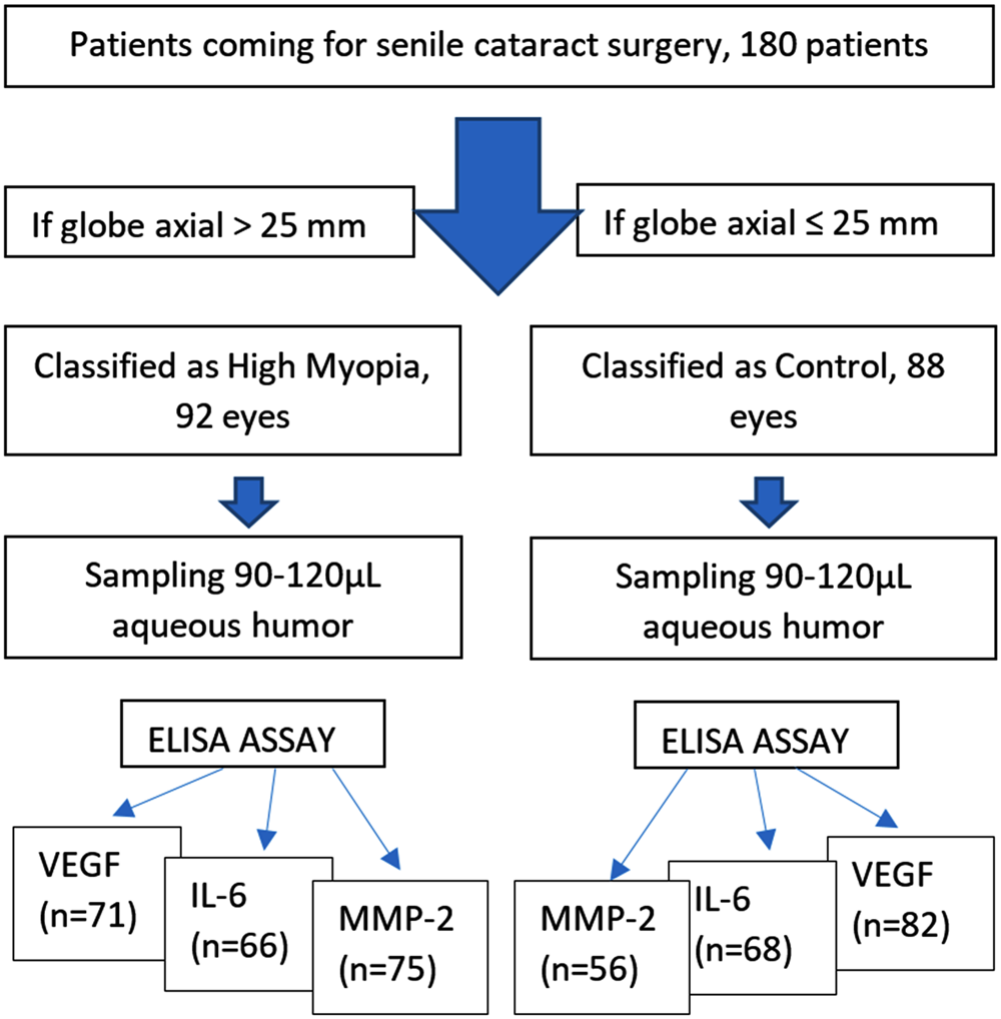
Study Flowchart.

Patients who underwent cataract surgery between January 2016 and October 2017 were recruited for this study as the study flowchart shown (Fig. 1). During the pre-surgical visit, patient medical history and family history were carefully discussed with the patients and reviewed systematically. Patients with any previous intraocular surgery, trauma, or any known eye diseases such as diabetic or high blood pressure retinopathy, a history of retinitis, uveitis, retinal vein occlusion, or any type of macular degeneration other than myopic macular degeneration were excluded. Patients who had a family history of glaucoma or a personal history of glaucoma, systemic diseases such as diabetes or rheumatic disorders, or usage of steroids were excluded from the study. If patients underwent cataract surgery in one eye previously, it had to have been performed at least 5 months before the current eye surgery. Eyes with myopic choroidal neovascularization were also excluded.

### Ophthalmic examinations and data recording

Prior to the surgery date, a comprehensive ophthalmic examination was conducted with a slit-lamp and indirect ophthalmoscope. All patients were subjected to careful evaluation of aqueous humor inflammatory cell grading according to the SUN working group^26^. Any patient with a grade of 0.5 or higher was excluded from the study. All qualified patients were subjected to myopic maculopathy evaluation and grading during pre-surgical exams or the day after the cataract extraction. Myopic maculopathy was graded into five categories (no myopic retinopathy 0, tessellated fundus 1, diffuse chorioretinal atrophy 2, patchy chorioretinal atrophy 3 and macular atrophy 4, according to the international photographic classification and grading system for myopic maculopathy^27^. The classification was confirmed or rectified by a post-surgical fundus examination. In addition, intraocular pressure (IOP) was measured by applanation tonometry. Corneal refraction was determined by an auto refractometer (HAAG-STREIT LS900, Swaziland). Biometry and axial length were measured using the IOL Master (Version 3.01; Carl Zeiss Meditec AG, Germany). B-scan ultrasonography (Accutome, Esaote MyLab50, Italy), cornea curvature, cornea endothelium imaging (Topcon SP-3000P, Japan), and optical coherence tomography (Topcon3D OCT-2000) were also conducted. The patients were prescribed 0.5% Levofloxacin eye drops and instructed to apply 6 times the day before surgery. No other medications or eye drops were used.

### Cytokine selection and ELISA quantitation

Various cytokines have been implicated in myopia progression in experimental studies, including interleukin 6 (IL6)^28^ and metalloproteinase-2 (MMP-2)^22^. Vascular endothelial growth factor (VEGF) has been shown to be low in myopic eyes^29,30^; however, VEGF in highly myopic eyes has not been well investigated. The hypothesis behind this study is that chronic subclinical inflammation may play a role in eye globe elongation, assuming that inflammation promotes the breakdown of the extracellular matrix of the sclera and results in axial elongation. Therefore, the pro-inflammatory cytokine, IL-6, and an enzyme involved in the degradation of extracellular matrix proteins, MMP-2, were selected as the outcome measures of this investigation. During the surgery, the aqueous samples were collected into a sterile tube with standard aseptic techniques and immediately stored at −80 °C until analysis. IL-6, MMP-2, and vascular endothelial growth factor A (VEGF-A) were quantitated by the enzyme-linked immunosorbent assay (ELISA). The assay was performed according to the manufacturer’s instruction (Human IL-6 Immunoassay/Catalog Number: VAL102, R&D Systems, Inc., Minneapolis, MN, USA) (Total MMP-2 Immunoassay/Catalog Number: MMP200, R&D Systems, Inc., Minneapolis, MN, USA) (Human VEGF Immunoassay/Catalog Number: VAL106, R&D Systems, Inc., Minneapolis, MN, USA). A standard curve was constructed from 0 pg/mL to 1000 pg/mL for VEGF, 0 pg/mL to 100 pg/mL for IL6, and 0 pg/mL to 32 ng/mL for MMP-2. The concentrations of these proteins in the aqueous humor were determined from the standard curve. If the samples were diluted to allow for the analysis of all three cytokines, the concentration read from the standard curve was multiplied by the dilution factor. To ensure accuracy, the dilution factor was kept under 4 and some aqueous samples were not enough for quantitation of all three cytokines. The minimum detection limit of VEGF is 7.8 pg/mL, 1.56 pg/mL for IL-6, and 0.033 ng/mL for MMP-2. If the concentration readouts are below the minimum detectable limits, the concentration numbers were derived from dividing the square root of 2 into the minimum detection limit.

### Statistical analysis

The continuous data was expressed as mean and standard deviation while the categorical data was expressed by fraction or percentage. Categorical data was compared between groups by Chi square or Fisher’s exact test. The data with normal distribution or transformed normalized data were analyzed with either t-test or multivariate linear regression for comparisons between the high myopia and the control groups while adjusting for gender, age, and IOP. A linear regression analysis using the globe axial length to predict the level of the cytokine in aqueous was also performed. All tests were two-tailed and the significance was considered statistical when p < 0.05.

## Results

### Baseline characteristics of the participants

**Figure 2:**
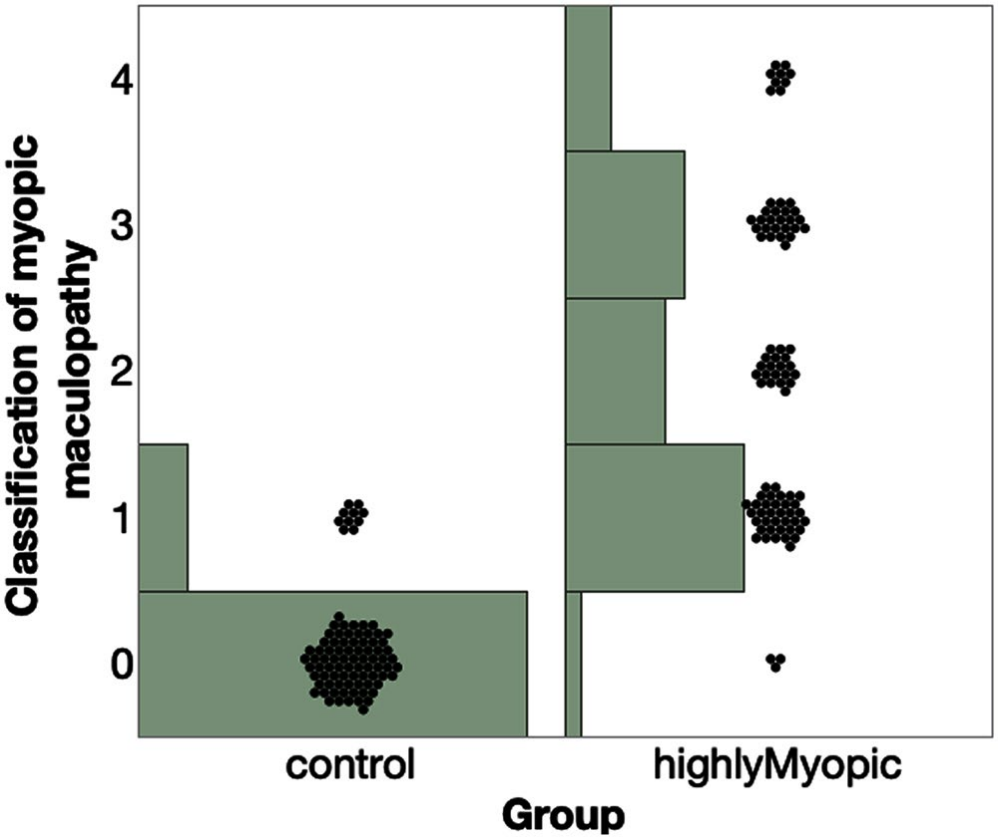
Distribution of myopic maculopathy within each group. 0 denotes a normal fundus, 1 denotes a tessellated fundus, 2 denotes diffuse chorioretinal atrophy, 3 denotes patchy chorioretinal atrophy, and 4 denotes macular atrophy.

We obtained 180 aqueous samples from 180 Chinese patients who were scheduled for senile cataract surgery. Out of 180 samples, 92 samples were from highly myopic eyes and the other 88 samples were from regular senile cataract patients without high myopia or myopia with a globe axial length no longer than 25 mm. The pre-operative distributions of age, gender, IOP, refractive spherical equivalent, depth of the anterior chamber, eye globe axial length, and cornea endothelium count are summarized in Table 1. The two groups did not differ by gender and cornea endothelium count. The other parameters (Table 1) were statistically different between the two groups. The globe axial length of highly myopic eyes was 26% longer (6.17 mm longer) than that of the concurrent control eyes (29.49 ± 2.25 vs. 23.32 ± 0.84, p < 0.0001 t-test). Out of 92 highly myopic patients, 73% had staphyloma while none of the control eye had staphyloma. The distribution of myopic maculopathy is presented in Fig. 2. Only 11% of the control eyes had tessellated fundus (grade 1) and the rest of the eyes had a normal fundus (grade 0). In contrast, only 3% of the highly myopic eyes had a normal fundus (grade 0): 39% with a tessellated fundus (grade 1), 22% with diffuse chorioretinal atrophy (grade 2), 26% with patchy chorioretinal atrophy (grade 3), and 10% with macular atrophy.

**Table 1:** Baseline characteristics of the patients by groups.

Group	Age	Gender	IOP (mmHg)	SE (diopter)	AC depth (mm)	AL (mm)	Cornea endothelium/mm^2^
Control	68.4 ± 8.7	F66%/M34%	14.7 ± 2.7	−1.15 ± 3.25	2.66 ± 0.37	23.32 ± 0.84	2561 ± 286
High Myopia	60.2 ± 9.8	F61%/M39%	15.6 ± 3.1	−14.78 ± 5.51	3.13 ± 0.48	29.49 ± 2.25	2592 ± 316
p value	<0.0001	0.48	0.029	<0.0001	<0.0001	<0.0001	0.51

SE: Spherical Equivalent; IOP = Intraocular Pressure; AC: Anterior Chamber.

AL: Axial length of the eye globe.

### IL-6 levels in the aqueous humor of the study eyes

**Figure 3:**
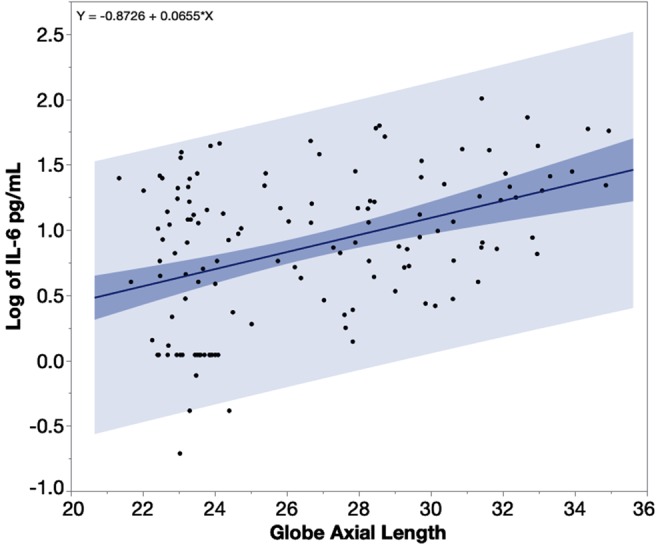
The data of IL-6 (Interleukin 6) was log-transformed to follow a normal distribution. IL-6 levels in the aqueous humor increase along the increase of globe axial length, with a numerical relation of 1.85 pg/mL increase per millimeter elongation of the studied eye globe with axial length between 22 to 36 mm. The heavier shaded area indicates the confidence region for the fitted line and the lighter shaded area indicates the confidence region for individual predicted values.

The multivariate linear regression revealed that IL-6 in the aqueous samples of the highly myopic eyes was significantly higher than in the aqueous of the control eyes (least square means 11.90 vs. 4.38 pg/mL, p < 0.0001) while adjusting for age, gender, and IOP. Neither the age (p = 0.84), gender (p = 0.45), nor IOP (0.53) was a significant factor. A linear regression of aqueous humor IL-6 levels over the eye globe axial length revealed a significant positive association between the two parameters (Fig. 3, β = 0.065, p < 0.0001, n = 134). For an eye with an axial length of 22 mm, the predicted IL-6 in the aqueous humor would be 3.63 pg/mL and 29.51 pg/mL for an eye with 36 mm of axial length. Each mm increase of the axial length led to 1.85 pg/mL increase of IL-6 in aqueous humor.

### MMP-2 levels in the aqueous humor of the study eyes

**Figure 4:**
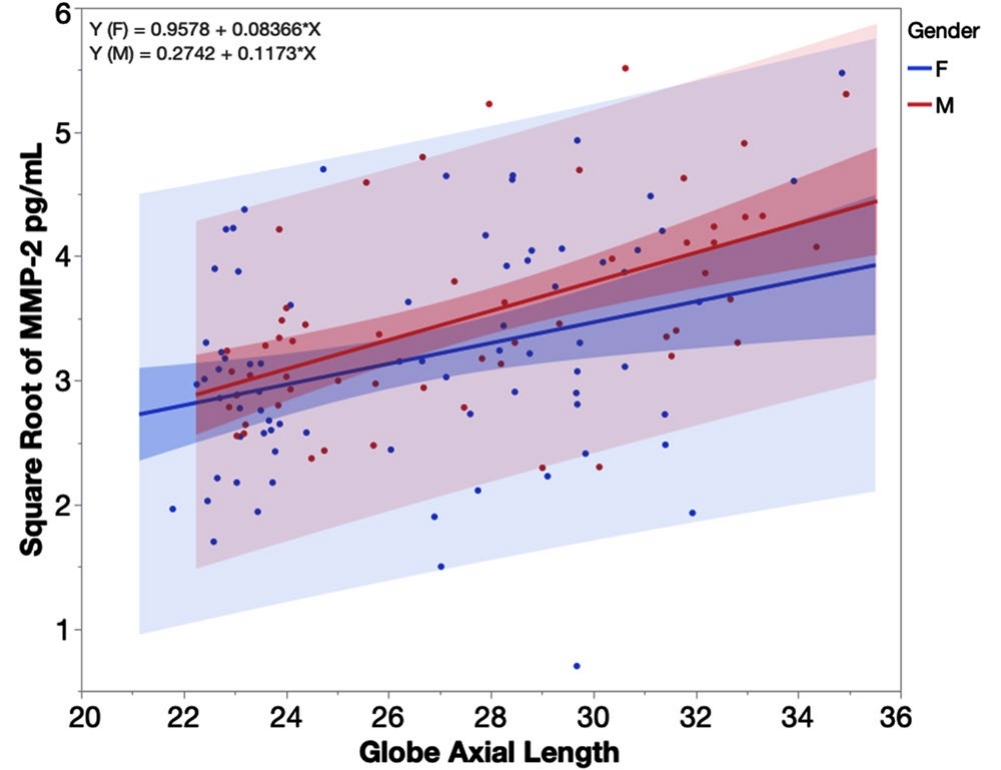
Regression plot for square root transformed MMP-2 (matrix metalloproteinase-2) by globe axial length stratified by gender. Blue line of fit for females (F) and red line of fit for males (M). Each mm increase in axial length of a female eye led to 0.57 ng/mL increase of MMP-2 in the aqueous humor while the increase was 0.86 ng/mL for a male eye within the axial range from 22 to 36 mm. The heavier shaded area indicates the confidence region for the fitted line and the lighter shaded area indicates the confidence region for individual predicted values.

The multivariate linear regression revealed that MMP-2 in the aqueous samples of the highly myopic eyes was significantly higher than in the aqueous of the control eyes (least square means 13.10 vs. 8.82 ng/mL, p = 0.0003) while adjusting for age, gender, and IOP. Age and IOP were not significant factors (p = 0.17 and p = 0.63), however, gender was a significant factor (F = 9.80 vs. M = 11.97, p = 0.029, n = 131). The regressions of aqueous humor MMP-2 levels over the eye globe axial length revealed significant positive associations between the two parameters (Fig. 4, p < 0.0001, n = 131) while adjusting for gender. For the eye of a female with an axial length of 22 mm, the predicted MMP-2 in the aqueous humor would be 7.83 ng/mL and 15.76 ng/mL for an eye with 36 mm of axial length. In contrast to the female, for the eye of a male with an axial length of 22 mm, the predicted MMP-2 in the aqueous humor would be 8.15 ng/mL and 20.22 ng/mL for an eye with an axial length of 36 mm. Each mm increase of the axial length of a female eye led to a 0.57 ng/mL increase of MMP-2 in the aqueous humor and a 0.86 ng/mL increase for a male eye.

### VEGF levels in the aqueous of the study eyes

**Figure 5:**
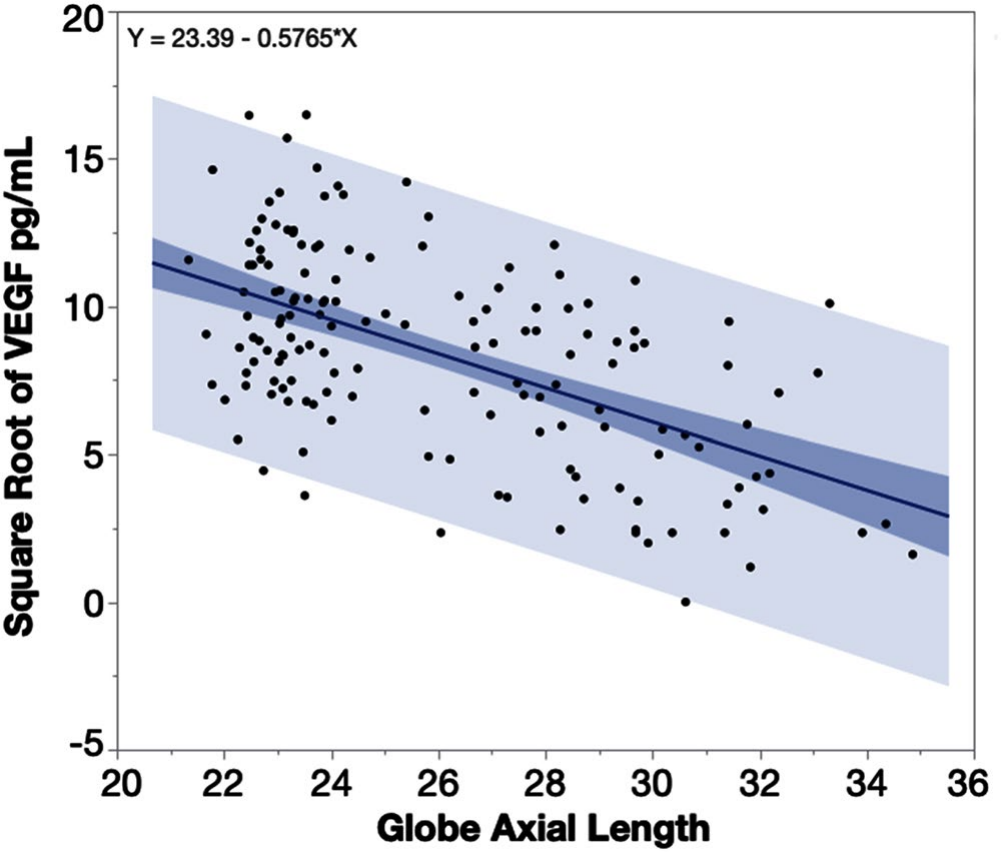
Plot of square root transformed aqueous humor VEGF (vascular endothelial growth factor) over the eye axial length. VEGF in the aqueous humor was negatively associated with the axial length of the eye globes. Each millimeter increase of axial length from 22 mm to 36 mm leads to a 5.54 pg/mL decrease of VEGF in the aqueous humor. The heavier shaded area indicates the confidence region of the fitted line and the lighter shaded area indicates the confidence region for individual predicted values.

The multivariate regression revealed that VEGF in the aqueous humor of the highly myopic eyes was significantly lower compared with the control eyes (least square means 45.56 vs. 96.90 pg/mL, p < 0.0001) while adjusting for age (p = 0.11), gender (p = 0.81), and IOP (p = 0.48). A linear regression analysis of aqueous VEGF and the globe axial length demonstrated that VEGF in aqueous humor was significantly associated with globe axial length in a negative mode (Fig. 5, p < 0.0001, n = 153). Each millimeter increase of axial length in a range of axial length 21 mm to 34 mm would lead to 5.54 pg/mL decrease of VEGF in aqueous humor.

### Sub-analysis of relationship between the cytokines in aqueous humor and the globe axial length in the eyes without retinal atrophic changes

Within this sub-analysis, the eyes with retinal atrophy ((diffuse chorioretinal atrophy (grade 2), patchy chorioretinal atrophy (grade 3), and macular atrophy (grade 4)) were excluded. The characteristics of the excluded patients were summarized in Table 2. The remaining patients had either a normal fundus or only a tessellated fundus, a very common finding in myopia. The regression of the cytokines on the eye globe axial length demonstrated the similar significant relationship as described above (for IL-6, β = 0.06, p = 0.0032; for MMP-2, β = 0.089, p = 0.002; for VEGF, β = −0.65, p < 0.0001). The average retina thickness within 1.5 mm (diameter) from the center of the macula had a tendency to be thinner for myopic group (ctrl = 234 vs. myopia = 217 µm, p = 0.09); and the macular volume was significantly smaller for myopia group (ctrl = 1157 vs. myopia = 1011 µm^3^, p < 0.0001) while adjusting for age and gender.

**Table 2:** Baseline characteristics of the patients with retinal atrophic change.

^#^Of patients	Age	Gender	IOP (mmHg)	SE (diopter)	AC depth (mm)	AL (mm)	Cornea endothelium/mm^2^
53	60 ± 9	F68%/M32%	15.3 ± 3.4	−15.98 ± 5.02	3.25 ± 0.46	30.44 ± 1.95	2561 ± 275

SE: Spherical Equivalent; IOP = Intraocular Pressure; AC: Anterior Chamber.

AL: Axial length of the eye globe.

## Discussion

Few studies have examined the possible association between chronic inflammation and myopia development or progression^21,22^. The association is largely based on retrospective clinical case observation^21,22,31^ or experimental animal data^22^. The mechanistic basis for the role of inflammation in myopia development and progression is not yet established. There is no tangible evidence that inflammation is indeed associated with high myopia in humans. One small sample study using human aqueous samples reported no significant association between eye axial length and inflammatory cytokines (IL-1β, IL6, and TGF-α) in 33 patients with an axial length in the range of 22.6–31.5 mm^29^. The study by Zhu *et al*. did not have a control group and the eyes included were emmetropic to mild or moderately myopic. The current study had a much larger sample size and eye axial lengths ranging from 21 mm to 35 mm, which revealed that inflammatory cytokines, IL-6 and MMP-2, are overexpressed in the ocular fluid of highly myopic eyes. We found a strong positive association between eye globe axial length and these two inflammatory cytokines. The data modeling suggests that each millimeter increase of globe axial length is associated with a 1.85 pg/mL increase of IL-6 and a 0.57–0.86 ng/mL increase of MMP-2 in the aqueous humor. Interestingly, the association between MMP-2 and eye globe axial length was stronger in males than in females.

Several molecules have been implicated in the development of myopia in experimental studies: MMP-2^32^, tissue inhibitor of metalloproteinase-2 (TIMP-2)^33^, and inflammatory cytokines including IL-6^22^. Lin *et al*.^22^ demonstrated the retinal levels of MMP-2 and IL-6 increased over time in the occluded eyes of guinea pigs. Rada *et al*.^33^ reported decreased expression of TIMP-2 in the sclera of form-deprived chick eyes while McBrien *et al*.^32^ reported increased scleral MMP-2 activity in tree shrew eyes during myopia development. It is assumed that signals originate from the retina and promote MMP-2 activity in the sclera, leading to a loss of extracellular matrix in the posterior sclera. The resultant thinning along with mechanical stress on the sclera allows for myopia development and progression^32,34–37^. However, if or how inflammatory cytokines play a role in human myopia is not yet clear.

Studies on human eyes similar to the studies done in animals are very limited due to poor availability of human eye tissue. It is simply not justified to collect the vitreous from high myopia patients. It is possible to sample the vitreous when such patients are under surgery for some eye diseases other than simple high myopic change; however, that eye disease or condition would bias cytokine profiles and make the study invalid. The current study is the most comprehensive so far and includes a large sample of human eyes. We took the opportunity during cataract extractions to sample clean aqueous humor without the influence of any eye diseases. Senile cataracts were present in all of the study patients. Though the degree of cataract was not graded in this study, a large sample size of this study may make disparity of cataract across the groups much less of a confounding factor. Although the aqueous is further away from the retina than the vitreous, studies have shown that drug and cytokine levels are closely associated in the same direction within these two ocular fluids^23,24^.

Other studies have indicated that cells in the retina, such as pigment epithelium, actively participate in the pathogenesis of myopia through biological signaling (cytokines)^38,39^. In animal models of myopia, higher MMP-2 activity was detected from the sclera^32^ and retina^22^. If increased expression of MMP-2 is seen in the retina of myopic animal eyes^22^, it is logical that elevated MMP-2 would also be detectable in the aqueous humor. In addition to the current study, a smaller study of human aqueous humor from cataract surgeries showed higher MMP-2 and TIMP-2 in myopic eyes^40^.

In contrast to MMP-2, which has been well portrayed in scleral remodeling and thinning during experimental myopia studies, IL-6 is seldom implicated in myopia. Very little experimental research has investigated IL-6 in the sclera or intraocular tissues of myopic model eyes^22^. Clinically, the current study is the first to demonstrate a connection between this inflammatory cytokine and highly myopic eyes. In our study, IL-6 in the aqueous humor is shown to increase with elongation of the eye globe, indicating a connection between inflammation and eye globe elongation. IL-6 is an inflammatory mediator and upregulated in many inflammatory eye diseases such as uveitis^41^, diabetic retinopathy^42^ and in the eyes of patients with systemic diseases such as lupus^43^. All of these inflammatory diseases were excluded from the current study. Recently, a retrospective clinical cohort study reported that the odds of developing myopia was significantly higher in populations of uveitis, diabetic retinopathy, and lupus patients when compared with age and sex-matched controls^22^. This indirect evidence in addition to the direct evidence from the current study suggests that inflammatory cytokine IL-6 in the retina may serve as a trigger to initiate MMP-2 activity in first the retina, then the sclera, causing progressive scleral remodeling and thinning. In human pathophysiology, IL-6 has been linked to increased MMP-2 production^44^, especially in neurodegenerative and neuroinflammatory states^45^. Retinal atrophy, either diffuse or patchy, in high myopia is a type of neurodegenerative change. Though no data available in literature to suggest a connection between retinal atrophy and inflammation such as higher level of IL-6 in ocular fluid, brain neurodegeneration have been reported to show significant higher IL-6 level in cerebrospinal fluid^46^ or peripheral blood^47^. The retina is located in the back of the eye and is an evagination of the brain and part of the central nervous system. Though there is no direct evidence in literature to associate myopia with neuroinflammatory disease such as Alzheimer disease, studies have shown that both retinal nerve fiber layer thinning and choroidal thinning were present in Alzheimer disease^48–50^. Thinning of retina and choroid is the hallmark of high myopia^51^. At the same time, some studies have also implicated VEGF as a neuroprotective factor and insufficient VEGF is involved in neurodegeneration^52,53^. VEGF has long been noted to be lower in myopic eyes^29,30,54–56^. Interpretation of this finding has no consensus and no clear assumption has been made about the connection between low VEGF and high myopia. Sawada *et al*. suggested lower VEGF levels in myopic eyes are due to less production by the retina, not due to dilution by the larger eye volume^30^. However, Hu *et al*. suggested that low VEGF is due to the larger intraocular volume of myopic eyes and the effect of dilution^56^. Of these studies, Sawada *et al*. had the largest sample size of 60 eyes^30^. The current study had a much larger sample size (180 eyes) and the findings confirmed the previous reports. However, we believe the mechanism for lower VEGF levels in myopic eyes may be more complex than the proposed diluting effect. We postulate that low VEGF in myopic eyes may be responsible for myopic retinal degeneration noted in various stages of myopia. Recent studies have further suggested that VEGF may play a neuroprotective role *in vitro* and *in vivo* in brain or retinal degeneration^57,58^. It has recently been reported that prolonged suppression of ocular VEGF by repeated intravitreal injection of anti-VEGF agents may promote chorioretinal geographic atrophy^59^. This is another layer of evidence that a certain level of VEGF in the retina is imperative to maintain the health of retinal neurons.

The current study highlights the possible connection between highly myopic eyes and low-grade or subclinical inflammation. As the study is cross sectional, a possible reversed causality can be argued; that myopic maculopathy results in higher IL-6 and MMP-2 and lower VEGF. However, our sub-analysis shows the same trend even when the cases with advanced myopic retinopathy are excluded. All these analyses were adjusted for age and gender since age is associated with anti-retinal immunity in general^60,61^. The sub-analysis suggests that these cytokines were elevated or lowered prior to myopic retinopathy and may be regarded as prelude. The details of the mechanisms or intracellular signaling of these cytokines in high myopic eyes needs to be further explored.

In summary, the current study suggests there is a connection between low-grade intraocular inflammation and highly myopic eyes. It is possible that persistent subclinical inflammation of the retina/choroid is the driving force behind the elevated MMP-2 activity in the retina and the sclera, subsequent scleral thinning, and progressive eye globe axial elongation. However, since the current study was cross-sectional, the results should not be over-interpreted. Longitudinal studies and more cross-sectional studies are needed to further shed light on this topic. Nonetheless, the current study has highlighted the need to investigate the inflammatory aspect of high myopia and has provided a possible new direction of research to investigate the root cause of high myopia and the associated myopic retinopathy^62^.

## Data Availability

The datasets used and/or analyzed during the current study are available from the corresponding author on reasonable request.
